# Supplementary material for: The Role of ATF3 in Neuronal Differentiation and Development of Neuronal Networks in Opossum Postnatal Cortical Cultures
Source: Int J Mol Sci. 2022 Apr 29;23(9):4964. doi: 10.3390/ijms23094964 (PMC9100162; doi:10.3390/ijms23094964)
Supplement: Supplementary file 1 [file ijms-23-04964-s001.zip › ijms-1681740-supplementary.pdf]

SUPPLEMENTARY INFORMATION

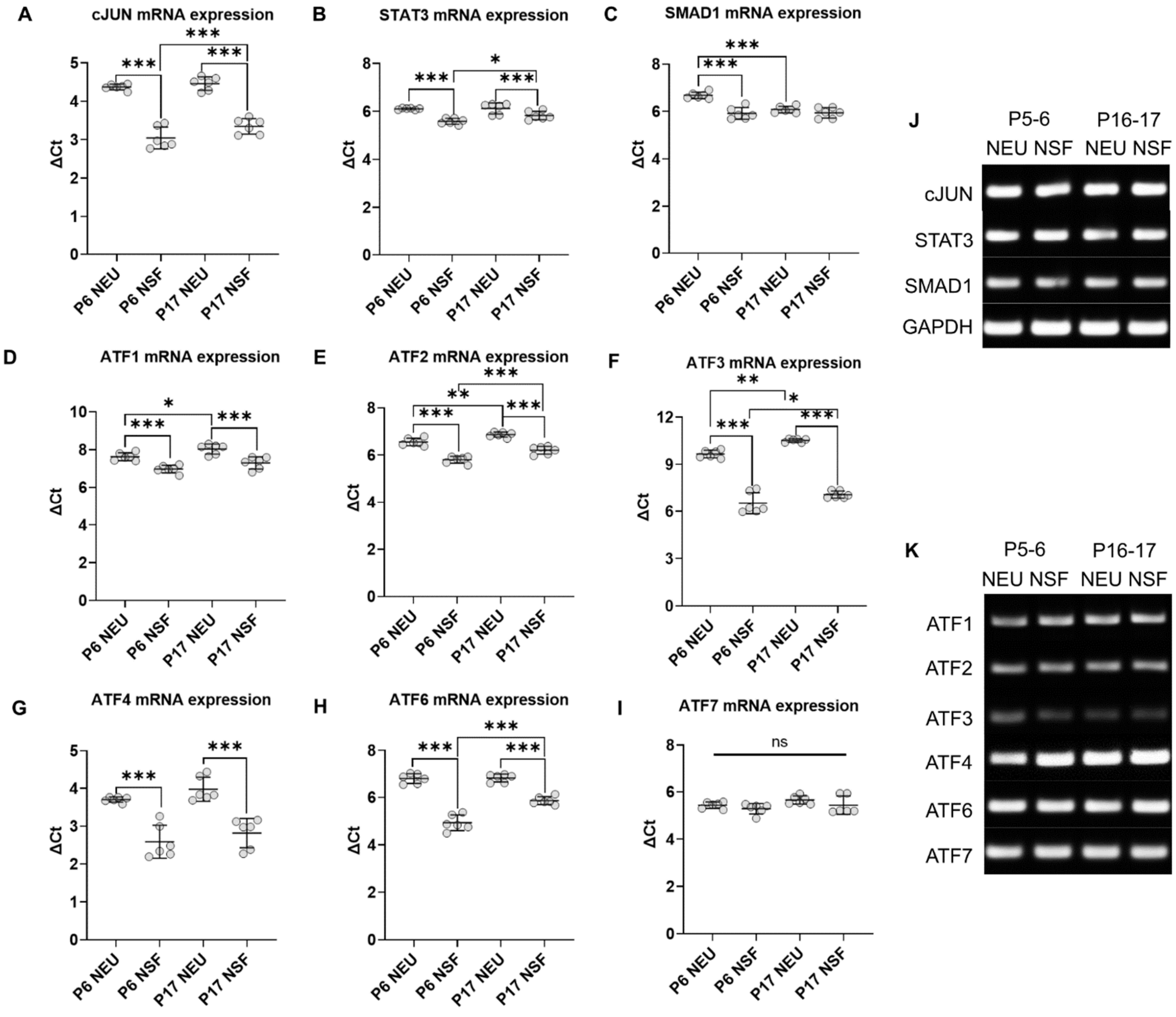

Supplementary Figure S1. Relative mRNA expression normalized to GAPDH (ΔCt). Scatter plots show mRNA levels measured by RT-PqCR, normalized to GAPDH, and expressed as ΔCt with mean ± SD. **(A)** cJUN: ANOVA with Holm-Šídák test, P6 NEU vs. P6 NSF $p < 0.001$***; P17 NEU vs. P17 NSF $p < 0.001$***; P6 NSF vs. P17 NSF $p = 0.03$*. **(B)** STAT3: ANOVA with Holm-Šídák test, P6 NEU vs. P6 NSF $p < 0.001$***; P17 NEU vs. P17 NSF $p = 0.02$*; P6 NSF vs. P17 NSF $p = 0.03$*. **(C)** SMAD1: ANOVA with Holm-Šídák test, P6 NEU vs. P6 NSF p <

0.001***; P6 NEU vs. P17 NEU p < 0.001***. **(D)** ATF1: ANOVA with Holm-Šídák test, P6 NEU vs. P6 NSF p < 0.001***; P17 NEU vs. P17 NSF p < 0.001***; P6 NEU vs. P17 NEU p = 0.03*. **(E)** ATF2: ANOVA with Holm-Šídák test, P6 NEU vs. P6 NSF p < 0.001***; P17 NEU vs. P17 NSF p < 0.001***; P6 NSF vs. P17 NSF p < 0.001***; P6 NEU vs. P17 NEU p = 0.001**. **(F)** ATF3: ANOVA with Holm-Šídák test, P6 NEU vs. P6 NSF p < 0.001***; P17 NEU vs. P17 NSF p < 0.001***; P6 NSF vs. P17 NSF p = 0.02*; P6 NEU vs. P17 NEU p = 0.002**. **(G)** ATF4: ANOVA with Holm-Šídák test, P6 NEU vs. P6 NSF p < 0.001***; P17 NEU vs. P17 NSF p < 0.001***. **(H)** ATF6: ANOVA with Holm-Šídák test, P6 NEU vs. P6 NSF p < 0.001***; P17 NEU vs. P17 NSF p < 0.001***; P6 NSF vs. P17 NSF p < 0.001***. **(J, K)** Agarose gel electrophoresis of endpoint RT-qPCR products**.** mRNA from P5-6 and P16-17 opossum cortical neurons and neurospheres. GAPDH is reference gene.

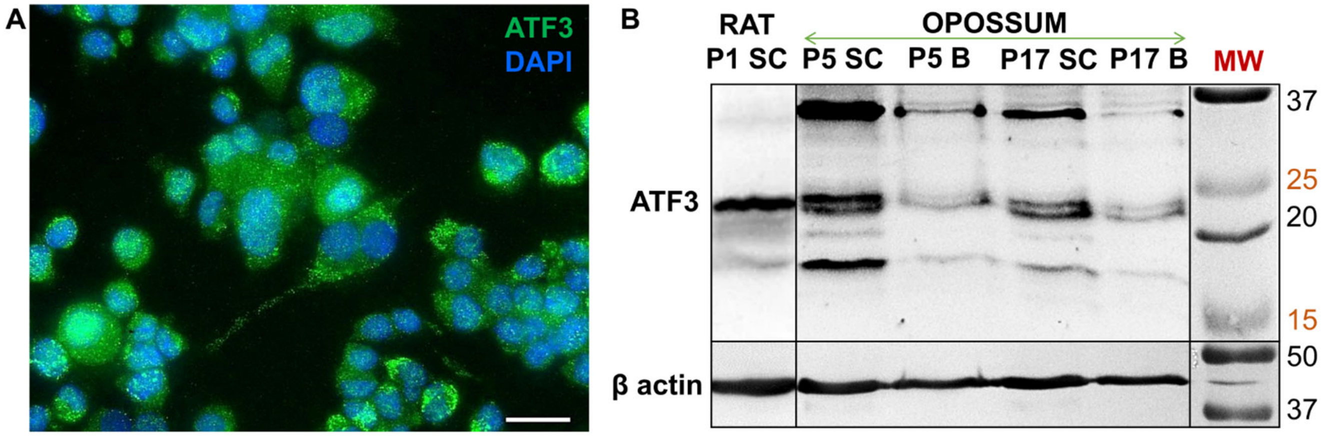

Supplementary Figure S2. Validation of the ATF3 antibodies used in this study for immunocytochemistry and western blot. (A) NSC34 cell line fixed at DIV7 and immunostained for ATF3 (green; ab216569). Cell nuclei were counterstained with DAPI (blue). Scale bar is 25

μm. **(B)** ATF3 (C-19; sc-188) antibody tested on the neonatal rat spinal cord tissue (P1 SC), and neonatal opossum P5 and P17 brain (B) and spinal cord (SC) tissue.

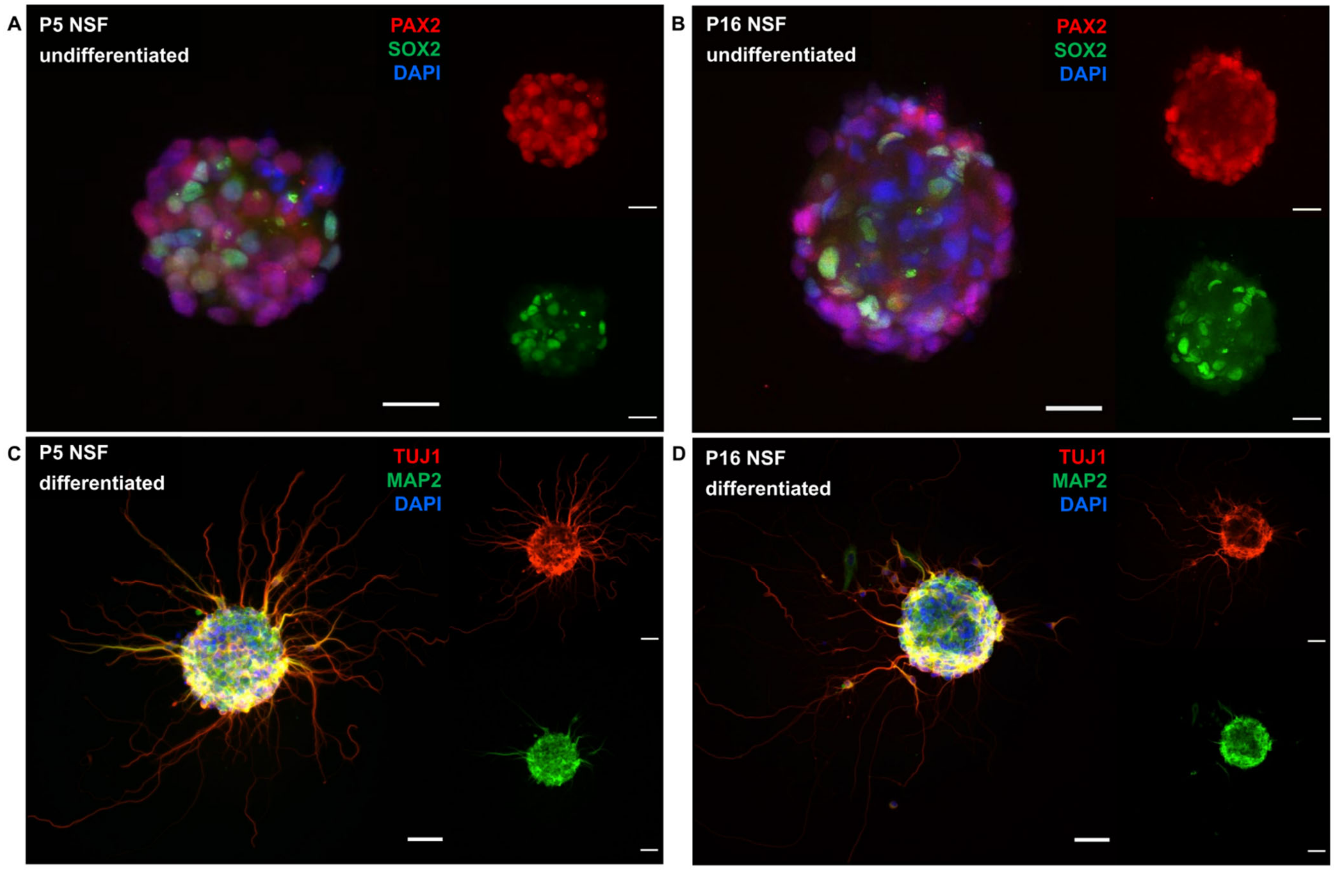

Supplementary Figure S3. Neurogenic potential of neurospheres. Neurospheres obtained from the cortex of neonatal opossums of different age groups, P5 **(A, C)** and **(B, D)** P16. P5 **(A)** and P16 **(B)** neurospheres were cultured in a proliferating media (DMEM/10%FBS) for a week. Neurospheres were fixed at DIV8 and immunostained for PAX2 (red), SOX2 (green) and nuclei were counterstained with DAPI (blue). Scale bar is 20 μm. P5 **(C)** and P16 **(D)** neurospheres were transferred at DIV8 on poly-L-ornithine/laminin coated coverslips in a neuronal medium to differentiate for the additional week. Neurospheres were fixed at DIV15 and immunostained for TUJ1 (red), MAP2 (green) and nuclei were counterstained with DAPI (blue). Scale bar is 50 μm.

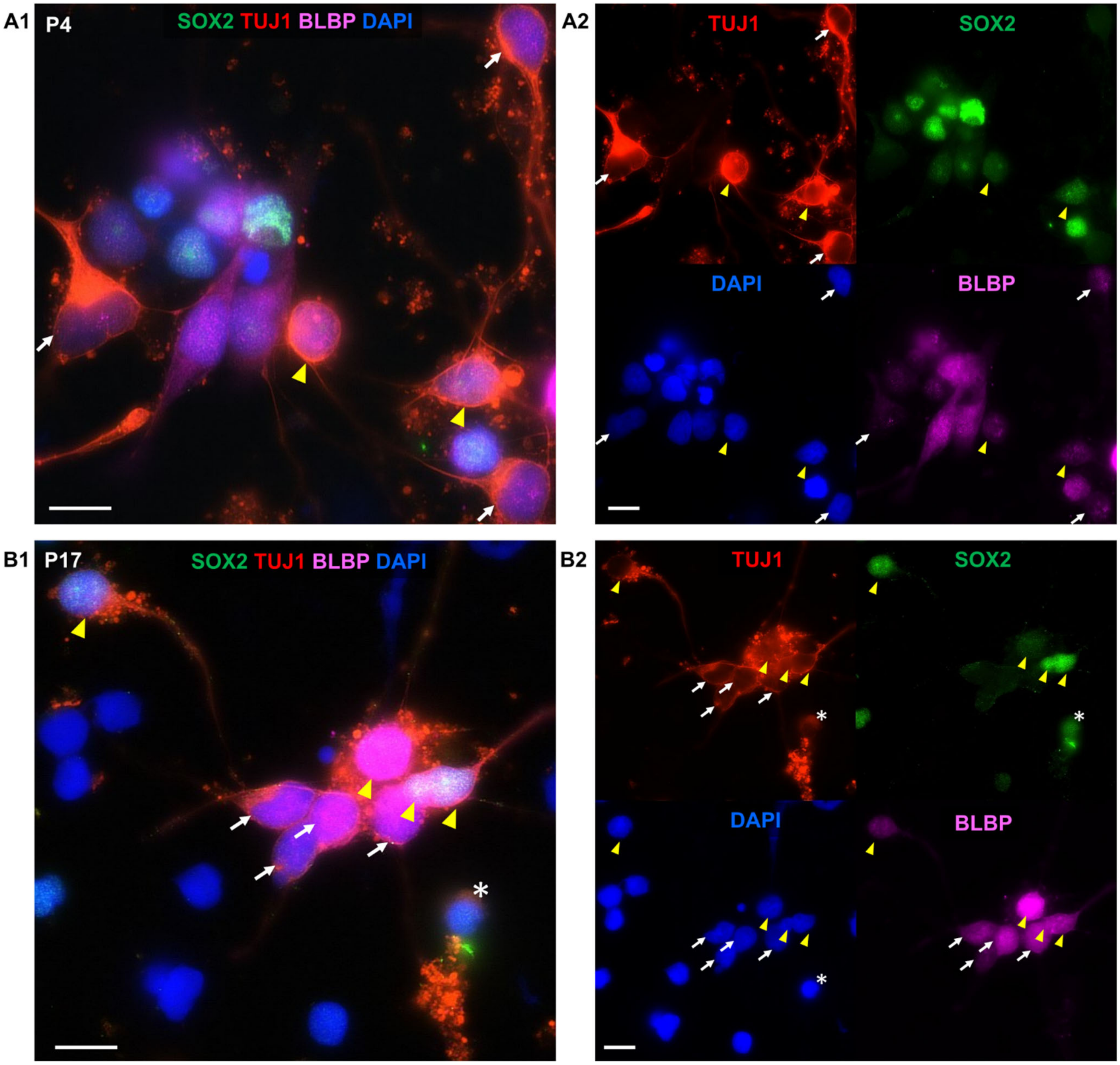

Supplementary Figure S4. Expression of neuronal progenitor markers. Cortical cultures are derived from P4 **(A1-2)** and P17 **(B1-2)** opossum. Cells were fixed at DIV2 and stained for TUJ1 (red), SOX2 (green), BLBP (magenta), and counterstained with DAPI nuclear stain (blue). Yellow arrowheads indicate $TUJ1^+/SOX2^+/BLBP^+$ neuronal progenitors, arrows indicate $TUJ1^+/SOX2^-$

/BLBP$^+$ neuronal progenitors, and asterisks indicate TUJ1$^+$/SOX2$^+$/ BLBP$^-$ neuronal progenitors. Scale bar is 10 μm.

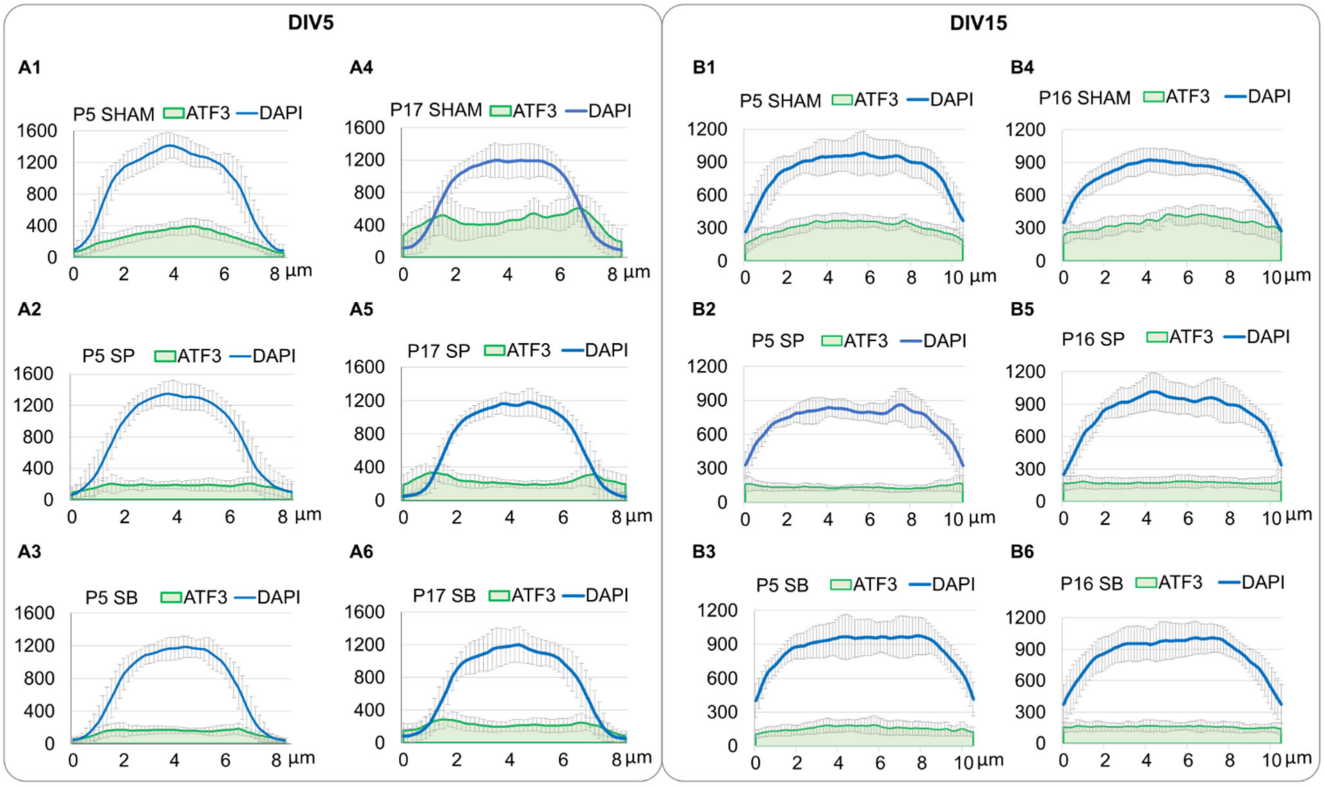

Supplementary Figure S5. Line scan analysis of ATF3 and DAPI average fluorescence intensity in primary neurons. (A1-A3) Line scan analysis of P5 primary neurons, and **(A4-A6)** P17 primary neurons at DIV5 for conditions: SHAM, SP and SB. **(B1-B3)** Line scan analysis of P5 primary neurons, and **(B4-B6)** P16 primary neurons at DIV15 for conditions: SHAM, SP and SB. Line scan analysis is performed on a Z-stack average intensity projection to obtain the plots in which ATF3 (green) and DAPI (blue) are shown (within the vertical lines on the plot is nucleus). The graph represents the average ATF3 and DAPI immunofluorescence intensity measured in 10-16 randomly selected neurons for each condition. Data are shown as mean ± SD.

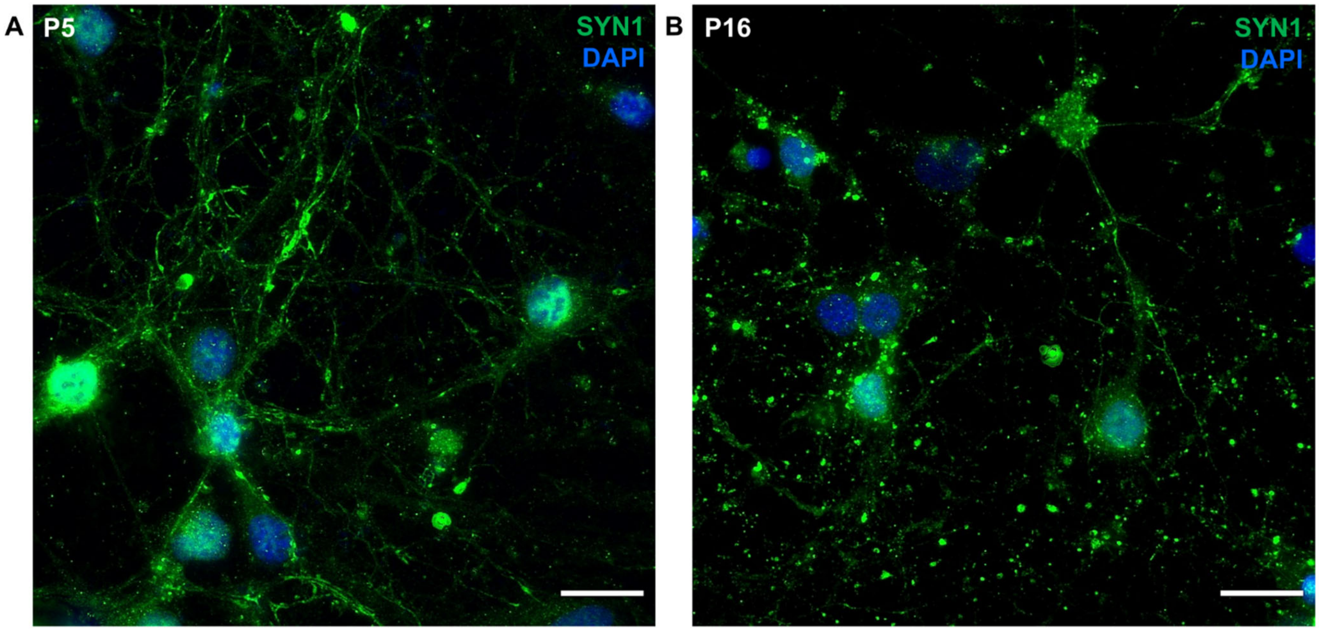

Supplementary Figure S6. Expression of Synapsin 1 in neuronal cultures after two weeks *in vitro*. P5 **(A)** and P16 **(B)** *M. domestica* primary cortical neuronal cultures were fixed at DIV15 and immunostained for Synapsin 1 (green). Cell nuclei were counterstained with DAPI (blue). Scale bar is 20 μm.

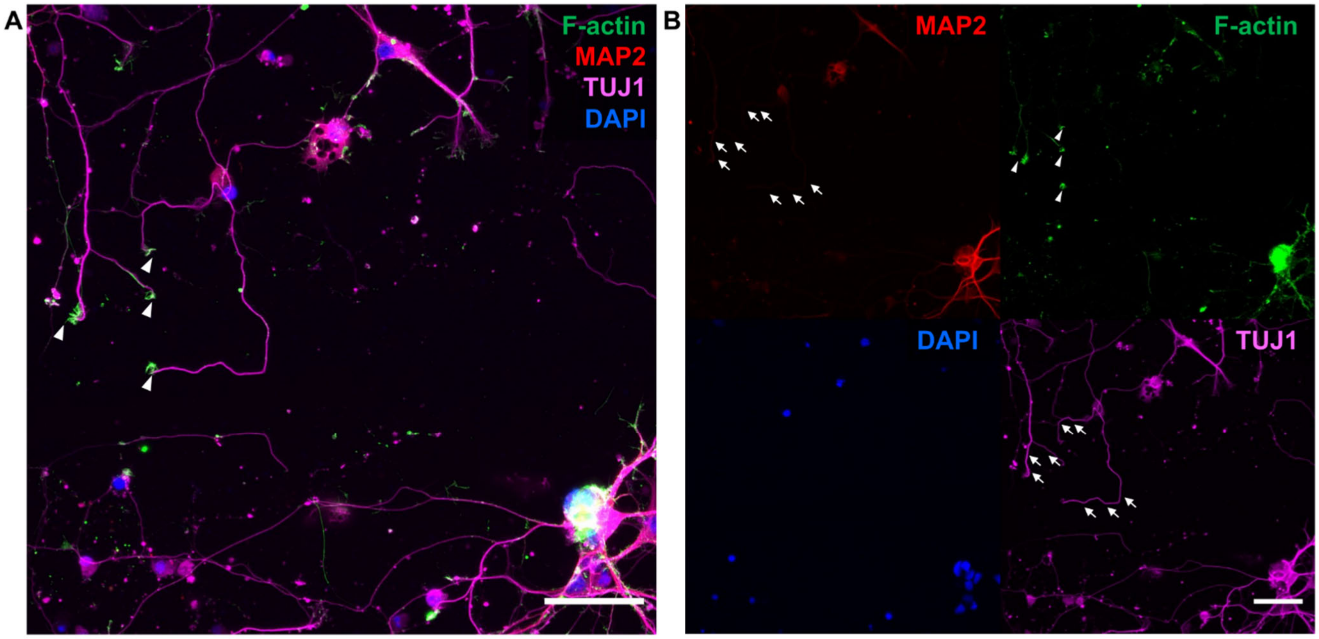

Supplementary Figure S7. Growth cones formation and axonal regeneration after injury.

(A, B) P5 opossum primary cortical neuronal cultures. Neurons were fixed after scratch at DIV16 and stained for MAP2 (red), F-actin (green), TUJ1 (magenta) and nuclei were counterstained with DAPI (blue). **(A)** Arrowheads indicate growth cones F-actin (green) after cut, stained with fluorescent phalloidin (Abcam, ab176753, 1:200). **(B)** White arrows indicate $TUJ1^+/MAP2^-$ regenerating axons.

Supplementary Table S1. Primary antibodies additional information

Antibody	Host and isotype	Dilution	Immunogen	Producer Catalog # RRID	Uniprot Align Immunogen Identity
**ATF3**	Rabbit polyclonal	1:100	C-terminus of ATF-3 of **human** origin	Santa Cruz Biotechnology sc-188 AB_2258513	92.3%
**ATF3**	Rabbit polyclonal	1:50	Synthetic peptide within **human** ATF3 aa 50-150	Abcam ab216569	92.3%
**BLBP**	Rabbit polyclonal	1:200	Synthetic peptide conjugated to KLH within residues 1 - 100 of **mouse** BLBP	Abcam ab32423 AB_880078	89.4%
**MAP2**	Mouse monoclonal IgG_1	1:200	**Bovine** MAP2	Sigma-Aldrich M1406 AB_477171	82.8%
**PAX2**	Rabbit monoclonal IgG	1:50	Synthetic peptide within **human** PAX2 aa 1-100	Abcam ab79389 GR3222251-1	94.5%
**SOX2**	Mouse monoclonal IgG_1	1:200	Synthetic peptide corresponding to **human** SOX2 aa 300 to the C-terminus conjugated to KLH	Abcam ab79351 AB_10710406	91.7%
**Synapsin 1**	Rabbit polyclonal	1:100	Synapsin I (mixture of Ia & Ib) purified from **bovine** brain.	Millipore AB1543 AB_2200400	72.3%
**β - Tubulin III (TUJ1)**	Mouse monoclonal IgG_{2a}	1:200	Microtubules derived from **rat** brain	Biolegend 801201 AB_2313773	99.8%
